# Report of methamphetamine use and cardiomyopathy in three patients

**DOI:** 10.1186/2008-2231-20-20

**Published:** 2012-08-30

**Authors:** Roxana Sadeghi, Khosro Agin, Maryam Taherkhani, Leila Najm-Afshar, Lewis S Nelson, Mohammad Abdollahi, Shahin Shadnia

**Affiliations:** 1 Internal Medicine Department, Loghman Hakim Hospital, Faculty of Medicine, Shahid Beheshti University of Medical Sciences, Tehran, Iran; 2 New York University School of Medicine, New York City Poison Control Center, Bellevue Hospital, New York, USA; 3 Faculty of Pharmacy, Pharmaceutical Research Center, Tehran University of Medical Sciences, Tehran, Iran; 4 Clinical Toxicology Department, Loghman Hakim Hospital Poison Center, Faculty of Medicine, and Toxicological Research Center, Shahid Beheshti University of Medical Sciences, Tehran, Iran; 5 Clinical Toxicology Department, Loghman Hakim Hospital Poison Center, Kamali Street, South Karegar Avenue, Tehran, 1333431151, Iran

**Keywords:** Cardiomyopathy, Congestive heart failure, Methamphetamine

## Abstract

**Background:**

Methamphetamine (meth) is a stimulant used illegally around the world, including in Iran. Cardiomyopathy and cardiac failure may occur following chronic meth use and may cause the patients referred to the emergency department.

**Case reports:**

A 28-year old man and two women, ages 29 and 31-year-old, with a history of meth use, were admitted to the emergency department with severe dyspnea at rest. Each had sinus tachycardia with tachypnea and an echocardiogram that showed severe systolic dysfunction consistent with heart failure. Additional evaluation in the hospital revealed cardiomyopathy with no other etiology other than the meth use.

**Conclusion:**

There are several reports that show an increase in frequency of meth use, suggesting that cardiomyopathy and acute heart failure may be a new medical concern.

## Background

Substance misuse is a major health problem in all parts of the world. Methamphetamine (meth) is a synthetic amine stimulant that is a highly addictive stimulant, and is currently the most widespread illegal amine drug used in the United States [1]. Its use has increased during the past two decades, especially in teenagers [2,3]. The last report by the Iranian drug control headquarters showed that only 3.6% of substance users in Iran used meth [4]. However, in recent years the local production of meth has risen and its price has decreased, resulting in growing use of the drug. Nonofficial reports estimate that meth is currently the second or third most widely used illicit substance in Iran [5].

Chronic use results in a number of medical complications and fatalities [6]. Meth directly affects multiple organs, as well as causes hypertension and tachycardia, cardiovascular complications such as myocardial infarction, dysrhythmias, ventricular hypertrophy, pulmonary edema and hypertension, cerebral stroke and hemorrhage, seizures, psychosis, and occasionally death may occur [7]. Due to the combination of some of these effects, long term users may develop cardiomyopathy. Although the incidence of methamphetamine cardiomyopathy is unknown, we report three cases of methamphetamine cardiomyopathy in Iran.

## Case reports

Three patients, a 28-year-old man, and a 29 and 31-year-old woman, were admitted separately in Loghman Hakim Hospital, a referral and tertiary care medical center in Tehran, Iran. All had a chief complain of severe dyspnea at rest. The 28-year-old man also complained of exertional chest pain which was typical ischemic chest pain. On arrival, all of them were conscious and followed commands. None of them had any underlying disease and the only pertinent positive in their medical history was meth use, which was supported by a urinary drug screen. The duration of meth use in the man was one year and was 2 and 3 years in the 29 and 31-year-old woman, respectively.

Vital signs on arrival included: BP: 90/70 mmHg, 100/60 mmHg and 90/60 mmHg, PR: 120, 104 and 110 beats/min and RR: 26, 32 and 22 breaths/min, respectively. A chest radiograph showed pulmonary congestion and an electrocardiogram (ECG) revealed sinus tachycardia, poor R wave progression in the pericardial leads, and T wave inversions in precordial leads in all three patients. Also, the second patient showed right axis deviation (135°) and the third patient had QS wave in the inferior leads. Measurement of cardiac troponin, evaluation of renal and thyroid function tests, measurement of serum electrolytes, blood lipids and a complete blood cell count (CBC) are shown in Table 1. A normal fasting transferrin saturation test was subsequently performed, ruled out hemochromatosis as an etiology of the cardiomyopathy.

**Table 1:** Laboratory data of the patients at admission in the hospital and during hospitalization

**31-year-old woman**	**29-year-old woman**	**28-year-old man**	**Laboratory parameter (Normal range)**
101 (1)	48 (1)	66 (1)	Urea (15–45 mg/dL)
1.5 (1)	1.0 (1)	1.5 (1)	Creatinine (0.7–1.4 mg/dL)
136 (1)	137 (1)	145 (1)	Na (135–150 mEq/L)
4.8 (1)	4.9 (1)	4.9 (1)	K (3.2–5.5 mEq/L)
8.5 (3)	9.0 (3)	9.9 (3)	Calcium (8.5–10.5 mg/dL)
5.1 (3)	4.6 (3)	4.4 (3)	Phosphorus (2.5–4.8 mg/dL)
2.1 (3)	2.0 (3)	1.9 (3)	Magnesium (1.9–2.5 mg/dL)
73 (1)	68 (1)	102 (1)	Creatinine phosphokinase (24–195 U/L)
15 (1)	8 (1)	16 (1)	CPK-MB (0–24 U/L)
14.1 (1)	14.7 (1)	12.6 (1)	Prothrombin time (12–14 second)
26.0 (1)	28.0 (1)	34.5 (1)	Partial thromboplastin time (24–36 second)
1.63 (1)	1.8 (1)	1.27 (1)	International normalized ratio (up to 1)
10700 (1)	17900 (1)	14100 (1)	White blood cell (4000–10000/μL)
11.9 (1)	8.1 (1)	12.4 (1)	Hemoglobin (12–14 g/dL)
34.3 (1)	28.5 (1)	39.9 (1)	Hematocrit (30–45%)
333000 (1)	215000 (1)	246000 (1)	Platelet (150000-350000/μL)
13 (3)	9 (3)	3 (2)	Erythrocyte sedimentation rate (0–10 mm/h)
Normal	Normal	Normal	Thyroid function tests
Negative	Negative	Negative	Human immune deficiency virus
106 (3)	104 (3)	248 (3)	Total cholesterol levels (mg/dL)
95 (3)	110 (3)	245 (3)	Serum triglycerides (mg/dL)
35 (3)	31 (3)	161 (3)	LDL cholesterol (mg/dL)
52 (3)	51 (3)	38 (3)	HDL cholesterol (mg/dL)
72 (1)	90 (1)	70 (2)	Fasting blood sugar (60–110 mg/dL)
0.4 (3)	ND	0.5 (3)	Troponin (adult < 1.3 mg/mL)

ND = Not determined.

The data in the parenthesis show the days after admission.

The 28-year-old man underwent coronary angiography due to concomitant chest pain, which was normal. All three patients underwent two-dimensional echocardiography with Doppler during the initial evaluation, and revealed severe decrease in left ventricular ejection fraction (LVEF) in each.

The patients were treated with angiotensin-converting enzyme inhibitors (ACEIs), beta-adrenergic antagonists, diuretics and digoxin and were discharged after 7, 10 and 20 days hospitalization. At the time of discharge, the 2 women were classified as having New York Heart Association class I (NYHA-I) and the man NYHA-III heart failure [8]. Outpatient visits were scheduled and the patients have been followed up for 9, 5 and 6 months respectively; no clinical or echocardiographic improvements were noted. The man patient was considered for heart transplantation due to his severe symptoms at rest despite optimal medical treatment.

Written informed consent was obtained from the patients for publication of this report and any accompanying images.

## Discussion

Meth is relatively easy to manufacture making its production inexpensive and widely available. Its low cost and long duration of action have made it a very desirable drug for use [5]. In addition, its high addictive potential and stimulant effects have made its use a serious health problem [9,10].

Meth affects multiple organs, including the cardiovascular system [7]. One report suggests that 40% of young patients with cardiomyopathy are meth abusers [11]. In another study, meth use was present in at least 5% of all patients presenting to the emergency department with heart failure [12]. Previous case reports and case series suggest that meth exposure is potentially associated with structural and functional changes of myocytes, as well as clinical manifestations of cardiomyopathy and congestive heart failure [13]. In Iran, the frequency of meth use especially within the young population has increased in the recent years [14,15], raising concerns for the future development of cardiomyopathy and acute decompensate heart failure in this group.

Diagnosing the etiology of dyspnea can be difficult, in part because several disorders may coexist. However, our patients were young and had no underlying medical problems except for meth use. The clinical evaluation found only left ventricular systolic dysfunction in all three patients. Although endomyocardial biopsies were not performed, the failure of clinical or echocardiographic improvement over time supports the diagnosis of meth-associated cardiomyopathy.

The most probably mechanisms for meth cardiotoxicity relates to the potent central and peripheral sympathomimetic effects of meth [16]. The increase in circulating catecholamine levels caused by this drug causes coronary vasospasm, persistent tachycardia and hypertension, and/or direct myocardial toxicity [17-19].

As in our cases, patients with meth-associated cardiomyopathy have a significantly lower LVEF or more severe ventricular dilation when compared with patients with cardiomyopathy from other causes [11,20]. As there is no antidote for the treatment of cardiac toxicity of meth use in various countries [21,22], some cardiac effects of its use, like myocyte hypertrophy and fibrosis are relatively irreversible [23], and cardiac toxicity may result in sudden and unexpected death [24]. By the way, recovery of left ventricular dysfunction in patients with meth-induced cardiomyopathy has been described [25], although recovery of cardiac function did not occur in any of our cases during follow up.

Recognition of cardiomyopathy and acute heart failure as a complication associated with the meth use may be a new medical concern.

## Competing interests

The authors declare that they have no competing interests.

## Authors’ contribution

All of the authors had the same contribution in article. All authors read and approved the final manuscript.
